# Karaya/Gellan-Gum-Based Bilayer Films Containing 3,3′-Diindolylmethane-Loaded Nanocapsules: A Promising Alternative to Melanoma Topical Treatment

**DOI:** 10.3390/pharmaceutics15092234

**Published:** 2023-08-29

**Authors:** Jéssica Brandão Reolon, Camila Parcianello Saccol, Bárbara Felin Osmari, Daiane Britto de Oliveira, Vinicius Costa Prado, Fernanda Licker Cabral, Lucas Saldanha da Rosa, Giancarlo Cervo Rechia, Daniela Bitencourt Rosa Leal, Letícia Cruz

**Affiliations:** 1 Laboratório de Tecnologia Farmacêutica, Programa de Pós-Graduação em Ciências Farmacêuticas, Centro de Ciências da Saúde, Universidade Federal de Santa Maria, Santa Maria 97105-900, RS, Brazil; jessica_breolon@yahoo.com.br (J.B.R.); camilapsaccol@gmail.com (C.P.S.); barbara_osmari@hotmail.com (B.F.O.); daianeoliveiraa125@gmail.com (D.B.d.O.); vini132007@gmail.com (V.C.P.); 2 Laboratório de Imunobiologia Experimental e Aplicada, Centro de Ciências da Saúde, Departamento de Microbiologia e Parasitologia, Universidade Federal de Santa Maria, Santa Maria 97105-9000, RS, Brazil; fernandalicker@hotmail.com (F.L.C.); daniela.leal@ufsm.br (D.B.R.L.); 3 Laboratório de Biomateriais, Centro de Ciências da Saúde, Departamento de Odontologia Restauradora, Universidade Federal de Santa Maria, Santa Maria 97015-372, RS, Brazil; lucas.saldanha@acad.ufsm.br; 4 Escola de Medicina, Universidade Franciscana, Santa Maria 97010-030, RS, Brazil; giancarlorechia@hotmail.com

**Keywords:** cutaneous delivery, indole-3-carbinol, skin cancer, solid formulations, nanocarriers

## Abstract

This study aimed to incorporate nanocapsules containing 3,3′-diindolylmethane (DIM) with antitumor activity into a bilayer film of karaya and gellan gums for use in topical melanoma therapy. Nanocarriers and films were prepared by interfacial deposition of the preformed polymer and solvent casting methods, respectively. Incorporating DIM into nanocapsules increased its antitumor potential against human melanoma cells (A-375) (IC_50_ > 24.00 µg/mL free DIM × 2.89 µg/mL nanocapsules). The films were transparent, hydrophilic (θ < 90°), had homogeneous thickness and weight, and had a DIM content of 106 µg/cm^2^. Radical ABTS^+^ scavenger assay showed that the DIM films presented promising antioxidant action. Remarkably, the films showed selective bioadhesive potential on the karaya gum side. Considering the mechanical analyses, the nanotechnology-based films presented appropriate behavior for cutaneous application and controlled DIM release profile, which could increase the residence time on the application site. Furthermore, the nanofilms were found to increase the permeation of DIM into the epidermis, where melanoma develops. Lastly, the films were non-hemolytic (hemolysis test) and non-irritant (HET-CAM assay). In summary, the combination of karaya and gellan gum in bilayer films that contain nanoencapsulated DIM has demonstrated potential in the topical treatment of melanoma and could serve as a viable option for administering DIM for cutaneous melanoma therapy.

## 1. Introduction

Cutaneous melanoma is a skin cancer originating in melanocytes, and its leading causes are genetic factors, skin phototypes (especially phototypes 1 and 2), and excessive exposure to solar radiation. Due to its high aggressiveness and metastatic rate, this form of skin neoplasm accounts for up to 80% of skin-cancer-related deaths [1,2,3]. Therefore, finding new therapeutic approaches for patients affected by cutaneous melanoma is crucial. Currently, surgical removal and the use of different antineoplastic drugs such as dacarbazine, temozolomide, paclitaxel, cisplatin, and monoclonal antibodies constitute therapeutic alternatives for melanoma [3,4]. However, therapy with the antineoplastics above can trigger a series of adverse effects, which can affect healthy cells and lead to a high degree of debilitation for the patient [3,4]. Therefore, novel approaches to enhance melanoma management must be prioritized.

3,3’-diindolylmethane (DIM) is a bioactive phytochemical sourced from cruciferous vegetables, and it has promising antitumor potential, including against melanoma [5,6,7,8,9]. Previous studies have demonstrated that DIM can modulate gene expression by inducing oxidative stress in melanoma cells, efficiently resulting in cycle arrest and cell apoptosis [7,9]. Despite the potential of DIM, its therapeutic use in a final pharmaceutical dosage form is hampered by unfavorable physicochemical characteristics. When administered orally, DIM’s low solubility in biological fluids and low permeability as a bioactive lead to reduced bioavailability [10,11]. Regarding parenteral administration, one of the main routes of administration of chemotherapy, the lipophilicity of DIM, can hinder possible formulations [12]. Another limitation of DIM is its chemical instability, especially in the light and at high temperatures [13,14,15].

Such limitations suggest that the cutaneous administration of DIM could be an alternative for treating melanoma. This approach involves using it directly on the skin, which can increase its concentration at the site of action and circumvent the limitations of the compound when administered via the systemic route. However, DIM clinical efficacy on the skin is limited since most compounds have inadequate physicochemical characteristics to overcome skin barriers, especially the stratum corneum [16,17]. Thus, the scientific community has investigated the potential of nanocarriers for the cutaneous delivery of anti-melanoma drugs, as they can increase the amount of active substance at the tumor site, overcoming the skin’s barriers and improving therapeutic effectiveness [18,19,20]. Polymeric nanocapsules are a nanostructured system of an oily core enveloped by a polymeric shell [21]. Lipophilic substances, such as DIM, tend to dissolve in the oil core, which can increase their solubility and encapsulation efficiency, enhancing their therapeutic effect. Thus, scientific evidence has demonstrated a greater encapsulation efficiency for DIM in polymeric nanocapsules when compared to its incorporation in other nanosystems, such as nanoparticles of poly (lactic-co-glycolic acid), zein, and isolated whey protein [13,14,15,22]. Furthermore, high encapsulation efficiency can enable the incorporation of lipophilic substances into polymeric films with hydrophilic properties and improve the photostability of compounds [15,23,24]. Different vegetable oils can create the oily core of these nanosystems, potentially enhancing the formulations’ therapeutic properties. In this context, pomegranate oil (*Punica granatum*), which contains abundant polyphenols and fatty acids, especially punicic acid, has been used for nanocapsule preparation and has presented relevant antitumor action [25,26,27,28].

Nanocarriers offer advantages for cutaneous delivery, including intimate contact with the skin, enhanced residence time, improved permeability, and sustained release of active substances to underlying layers [18,29,30,31]. Thus, nanocapsules containing anti-melanoma substances for use on the skin have been developed for these reasons [18,31,32]. Even though nanocapsules are acquired as an aqueous suspension, they can conveniently convert into other dosage forms suitable for their skin application, such as hydrogels and films [24,31]. Films are solid dosage forms with the potential for cutaneous application due to their ease of use, flexibility, and pleasant contact with tissues [33,34]. In addition, compared to semi-solid dosage forms, films can have greater dose accuracy and a lower frequency of administration [35]. Structurally, films can be mono- or multilayer, with multilayer films attracting interest because they preserve the properties of each film-forming agent in their respective polymeric layer, which can improve the overall properties of the film obtained [34,36,37].

Natural gums stand out among the polymeric materials used to prepare films since they are biocompatible, biodegradable, and non-toxic polymers [38]. Gellan gum, an extracellular polysaccharide obtained by aerobic fermentation of bacteria (*Pseudomonas elodea*), has been used to prepare mono- or bilayer films containing free or nanoencapsulated drugs for the topical therapy of cutaneous wounds, atopic dermatitis, and oral cancer [37,39,40]. Notably, gellan gum films usually present feasible characteristics such as mechanical strength, high swelling, and low water loss [24,37,41]. Another polysaccharide that stands out is karaya gum, obtained from exudative lesions on the stem of *Sterculia urens*, a tree typical of India and Africa [42,43]. Although this gum has essential properties such as bioadhesion behavior and control of the release of substances, few studies have been dedicated to preparing films of karaya gum for pharmaceutical purposes [44,45,46,47]. Furthermore, using karaya gum in developing these films was associated with other polymeric materials, in its deacetylated or cross-linked form, with no incorporation of nanostructured systems. Thus, creating a nano-based bilayer film consisting of gellan and karaya gum in distinct layers allows for the maintenance and proper investigation of the intrinsic properties of karaya gum as a film-forming agent. It is relevant to mention that there are currently no scientific reports on the advancement of films incorporating free DIM or nanocarriers with this bioactive.

In this context, in this study, we considered: (1) the need for new therapeutic approaches for melanoma, (2) the antitumor effect of DIM and its physicochemical limitations, (3) the benefits of associating this bioactive with nanocarriers, and (4) the advantages of using films for skin disorders. Our study aimed to incorporate DIM-loaded pomegranate oil nanocapsules in bilayer films consisting of gellan and karaya gums, to obtain an innovative therapeutic platform with potential application in the therapy of cutaneous melanoma.

## 2. Materials and Methods

### 2.1. Reagents

DIM (99.1% purity) was obtained from Fagron (São Paulo, Brazil). Span^®^ 80 (sorbitan monooleate), 2,2′-azinobis-(3-ethylbenzothiazoline-6-sulfonic acid) (ABTS), 3(4,5-dimethyl)-2,5diphenyltetrazolium bromide (MTT), Dulbecco’s Modified Eagle’s Medium (DMEM), penicillin/streptomycin, 0.25% trypsin/EDTA solution, and fetal bovine serum (FBS) were obtained from Sigma-Aldrich Co. (San Luis, MO, USA). Pomegranate oil was supplied by Florien (Piracicaba, SP, Brazil). Ethylcellulose was donated by Colorcon (Cotia, Brazil). Pullulan was donated by Hayashibara (Okayama, Japan). Polyethylene glycol (PEG 400) was acquired from Labimpex (São Paulo, SP, Brazil). Glycerol was obtained from Nova Química do Sul (Porto Alegre, RS, Brazil). Gellan gum low acyl (Kelcogel^®^) was donated by CP Kelco (Atlanta, GA, USA). Karaya gum was presented by Metachem (São Paulo, SP, Brazil). All other chemicals and solvents were analytical grade and used as received.

### 2.2. Analytical Procedures

DIM was quantified in nanocapsule suspensions and films using high-performance liquid chromatography (HPLC), following a methodology validated in our research group [15]. Chromatographic instruments and conditions were as follows: LC-10A HPLC system (Shimadzu, Kyoto, Japan) equipped with an SIL-20A HT valve sample automatic injector, a UV–VIS SPD-M20A detector, an LC-20AT pump, a CBM-20A system controller, a guard column, and a Kinetex C_18_ Phenomenex column (250 mm × 4.60 mm, 5 µm; 100 Å).

### 2.3. Preparation and Characterization of Nanocapsule Suspensions

Nanocapsule suspensions were prepared by interfacial deposition of the preformed polymer (*n* = 3) [48]. Initially, an organic phase consisting of ethylcellulose (0.1 g), Span 80^®^ (0.077 g), pomegranate oil (0.150 g), DIM (0.01 g), and acetone (50 mL) was obtained by solubilization under moderate magnetic stirring and heating at 40 °C for 60 min. Subsequently, the organic phase was injected into an aqueous phase consisting of pullulan (0.077 g) and distilled water (50 mL), and the mixture was kept under magnetic stirring for 10 min. Next, the organic solvent and excess water were removed using a rotary evaporator until 10 mL was reached (corresponding to 1 mg/mL of DIM). The obtained formulations were named NC-DIM. For comparative purposes, placebo formulations were also prepared (NC-B).

After preparation, the nanocapsule suspensions were evaluated for pH using a previously calibrated potentiometer (Model pH 140, Simpla, Sao Jose, Brazil). The formulations were also evaluated for particle size and polydispersity index (PDI) by dynamic light scattering and zeta potential by capillary electrophoresis (Zetasizer^®^ Nano-ZS ZEN 3600 model, Malvern Instruments, Malvern, UK). For this, nanocapsules were previously diluted (1:500) in ultrapure water or 10 mM NaCl solution before the analysis. To evaluate the DIM content in the formulations, an aliquot of 100 µL was placed in volumetric flasks (10 mL) and extracted with 3 mL of ultrapure water in an ultrasonic bath (Ultrasonic bath Q3.0/40A model, Ultronique, Indaiatuba, Brazil) for 15 min. Then, the volumetric flasks were filled with methanol and submitted again for extraction in an ultrasonic bath (15 min). Lastly, the samples were filtered (0.45 µm nylon membrane) and analyzed by HPLC (Section 2.2). Encapsulation efficiency (EE%) was evaluated by ultrafiltration/centrifugation. An aliquot (300 µL) of nanocapsules was placed in a 10,000 MW centrifugal filter device (Amicon^®^ Ultra, Millipore, Billerica, MA, USA), and the free bioactive fraction was separated from the nanocarriers at 2200× *g* for 30 min. Non-encapsulated DIM was determined in the ultrafiltrate by HPLC (Section 2.2), while the entrapped active compound was calculated according to Equation (1).

(1) $$EE\%=\frac{Total\;DIM\;content-Free\;DIM\;content}{Total\;DIM\;content}\times 100$$

### 2.4. In Vitro Antitumoral Assay

The in vitro antitumor action of DIM and its nanoencapsulated form was tested against human melanoma cells (A375), and cell viability was determined by 3(4,5-dimethyl)-2,5diphenyltetrazolium bromide assay (MTT) [49]. The A375 cells were obtained from Banco de células do Rio de Janeiro (BCRJ). The cells were grown and maintained in low-glucose Dulbecco’s Modified Eagle’s Medium (DMEM), supplemented with 10% (*v*/*v*) fetal bovine serum (FBS) and penicillin/streptomycin (100 U/L), in a humidified atmosphere of 5% CO_2_ at 37 °C.

To evaluate the in vitro antitumor effect, A375 cells were seeded in 96-well plates at a concentration of 4 × 10^5^ cells per well. These plates were kept at 37 °C in a humidified atmosphere of 5% CO_2_ for 24 h and then used for the tests. Then, the A375 cells were treated with nanocapsules (NC-DIM and NC-B) previously diluted in DMEM to reach the desired concentrations (corresponding to 1, 2, 4, 6, 12, 18, and 24 µg/mL of DIM). The concentration-curve range tested was selected based on studies demonstrating DIM antitumor action [15]. Free DIM was dissolved in dimethylsulfoxide (DMSO), followed by dilution in DMEM, respecting the maximum concentration of 0.01% (*v*/*v*) of DMSO in the medium. For comparative purposes, DMEM was also tested (negative control). Cells were incubated with the different treatments for 24 h and then incubated with 20 μL of MTT for 4 h at 37 °C. The formazan crystals formed were solubilized using 200 µL of DMSO and measured as an absorbance at 570 nm. The assays were performed in quadruplicate, and the cell viability of the treated groups was calculated and compared to the control culture with DMEM (negative control), representing maximum viability (100%). Furthermore, free or nanoencapsulated DIM values to reduce cell viability by 50% (IC_50_) were calculated in GraphPad Prism^®^ software (version 6).

### 2.5. Preparation of Bilayer Films

The films were prepared by a two-step solvent casting method [37]. First, an aqueous dispersion (25 mL) of gellan gum (0.1875 g) was obtained in a heating bath (80 °C) under magnetic stirring for 2 h. After complete dispersion of the polymeric material, 0.75 g of glycerol (3% m/V) was added under stirring to this dispersion. Then, this dispersion was poured into a petri dish (90 mm in diameter × 13 mm in height) and partially dried at 35 °C for 20 h. Subsequently, an aqueous dispersion (15 mL) of karaya gum (0.1875 g) was obtained in a heating bath (40 °C) under magnetic stirring for 1 h. After complete dispersion of the gum, 10 mL of nanocapsules (NC-DIM or NC-B) and 0.250 g of glycerol (1% *w*/*v*) were added, and this mixture was kept under magnetic stirring for 15 min. Then, this dispersion was deposited in Petri dishes over the first layer of gellan gum and completely dried in an oven at 40 °C for 24 h, thus obtaining bilayer films based on nanocapsules (F-NC-DIM and F-NC-B). Vehicle film (F-vehicle) was prepared for comparative purposes by adding distilled water instead of nanocapsule suspensions. In addition, a film containing free DIM (F-DIM) was also prepared, for which the bioactive (10 mg) was solubilized in 2.5 mL polyethylene glycol 400 (PEG 400) under magnetic stirring. After complete DIM solubilization, distilled water (7.5 mL) and glycerol (0.250 g) were added under magnetic stirring. Finally, this dispersion was added to Petri dishes containing the first layer of gellan gum, and the films were dried in an oven at 40 °C for 24 h.

### 2.6. Films Characterization

The films were characterized by transparency, DIM content homogeneity, weight homogeneity, thickness, and particle size (*n* = 3). The transparency of the films was determined with a spectrophotometer. The films were cut (0.5 cm base × 2.0 cm in height) in size sufficient to be placed in quartz cuvettes, then a spectrum scan (200 to 800 nm) was performed. To determine the homogeneity of DIM content, fragments of 1.0 cm^2^ were obtained in 3 different locations of each film. To extract DIM from the fragments, they were placed in a volumetric flask (10 mL) containing 3 mL of ultrapure water and submitted to an ultrasound bath (20 min). Subsequently, methanol was added to the volumetric flask, which was taken to the ultrasound bath (20 min) for complete extraction of the DIM. The samples were centrifuged (3000 rpm, 15 min) and filtered (0.45 μm), and the DIM content was determined by HPLC (Section 2.2). The average values obtained were expressed in µg/cm^2^, and the content (%) was calculated in relation to the theoretical amount of DIM present in the film (106 μg/cm^2^ corresponds to 100%).

Weight homogeneity was determined by weighing three fragments (1.0 cm^2^) obtained from different film regions on an analytical balance. The data obtained were expressed in mg/cm^2^. The films’ thickness was determined with a stereomicroscope (Discovery V20, Carl Zeiss, Gottingen, Germany), performing five measurements at different locations on the film. Mean thickness values were expressed in µm. To evaluate the presence of polymeric nanocapsules in the films, the particle size was determined by photon correlation spectroscopy. Therefore, the films (0.1 g) were redispersed in ultrapure water (50 mL) under magnetic stirring (1 h). After complete dispersion, the samples were centrifuged (3000 rpm for 15 min), filtered through a cellulose membrane (1.4 μm), and analyzed in a Zetasizer^®^.

### 2.7. Scanning Electron Microscopy

The morphology of films was evaluated by scanning electron microscopy (MEV; TESCAN^®^, model VEGA 3). The films were cryofractured after immersion in liquid nitrogen to visualize the layers, and analyzed in lateral sections after a fracture. The samples were previously covered with gold and analyzed using an accelerating voltage of 15 kV.

### 2.8. Mechanical Properties

The universal testing machine (Emic, São José dos Pinhais, Brazil) was utilized to determine the mechanical properties—tensile strength, deformation, and Young’s modulus—following the ASTM-D882-02 standards [50]. To conduct the tests, film samples with 60 mm × 45 mm dimensions were individually fixed to the machine probe. A tensile load was then applied at a 50 mm/min speed. The film’s maximum deformation was quantified by calculating the percentage change in sample length relative to its original size. Tensile strength was assessed by dividing the force required to rupture the film by the cross-sectional area of the strip. Meanwhile, Young’s modulus was determined by evaluating the ratio between stress and strain values. The results were expressed in MPa (tensile strength and Young’s modulus) and percentage (elongation).

### 2.9. Water Contact Angle

To assess the hydrophobicity of the films, the contact angle of water droplets on the film’s surface was examined using a goniometer (Drop Shape Analysis, DAS 30S model, Kruss; Hamburg, Germany). The film samples were cut into 2 cm × 1 cm fragments, and then 11 μL of distilled water was carefully added to the surface of each specimen using a micro-syringe. Subsequently, digital images were captured using a camera, and the contact angle was calculated as the angle formed between the tangent line on the droplet at the point of contact and the line drawn along the film’s surface. This calculation was performed in specialized software (DSA4 model) within 5 s of drop deposition. Polymeric films were evaluated in both layers (karaya or gellan gum), which were replaced at each reading. The results were expressed as mean measurement angle (θ) and mean standard deviation (*n* = 3).

### 2.10. Swelling Index

The determination of the swelling index was verified according to the methodology of Parodi et al. (2017) [51]. The films were cut (1 cm^2^) and placed in Petri dishes (40 mm), which were weighed. Subsequently, 6 mL of phosphate buffer pH 7.4 (PBS pH 7.4) was added, keeping the film fragment submerged. After 24 h, excess PBS 7.4 was carefully removed using an automatic pipettor, and the plate was weighed again. The swelling index was calculated according to Equation (2).

(2) $$Swelling\;index=\frac{WS-WD}{WD}\times 100$$

where WS is the weight of the film after swelling and WD is the weight of the dried film.

### 2.11. Bioadhesive Strength

The bioadhesive strength was determined by the methodology described by Osmari et al. (2020) [52], using an apparatus composed of two balanced arms. For this test, human skin obtained from discarded abdominoplasty surgery in female patients was used, an activity approved by the Research Ethics Committee of the Federal University of Santa Maria (CAAE: 27168719.4.0000.5346). After obtaining the skin, the adipose tissue was removed and frozen (−20 °C) until use. The skin was affixed to a glass plate beneath the frame. The films were brought into contact with the skin fragment by applying a force of 1 N for 60 s. Subsequently, water was steadily introduced through a plastic tube on the opposite side until the separation between the skin and film was observed. Then, the volume of water employed was measured using a graduated cylinder. The bilayer films’ upper layer (karaya gum) and the lower layer (gellan gum) were analyzed. The bioadhesive strength was determined using Equation (3) and expressed in units of dyne/cm^2^.

(3) $$Bioadhesive\;strength=\frac{\left(V\times G\right)}{A}$$

where V is the amount of water (g) required for the detachment between the sample and the tissue, G is the acceleration of gravity (980 cm/s^2^), and A is the area of exposed tissue (cm^2^).

### 2.12. In Vitro DIM Release

The in vitro release profile of DIM from films was determined using Franz diffusion cells. The diffusion area was 3.14 cm^2^, and a dialysis membrane (10,000 Da, Sigma Aldrich, Saint Louis, MO, USA) was placed between the donor and receptor compartments. The receptor compartment was filled with PBS pH 7.4 and ethanol (70:30), maintained at 32 ± 0.5 °C, and moderate agitation. Film fragments (1.0 cm^2^—corresponding to 106 µg/cm^2^) were placed on the membrane and hydrated with 200 µL of PBS pH 7.4. This pH value of the medium was defined as adequate to allow quantification of the bioactive in previous studies by our research group [15,53]. At times corresponding to 1, 2, 4, 6, 8, 10, and 12 h, aliquots of 200 µL of the receptor medium were withdrawn and replaced in the same volume. The amount of DIM transferred to the medium over time was determined by HPLC (Section 2.2). Experimental data were evaluated for DIM release kinetics, with data adjusted for zero order (*C* = *C*_0_ − *k* · *t*), first order (*LnC* = *LnC*_0_ − *k* · *t*), and second order (1/*C* = *k* · *t* + 1/*C*_0_) equations. Furthermore, the release mechanism was investigated using the Higuchi model (*Ct* = *k* · *t*^0.5^).

### 2.13. DIM Skin Permeation/Penetration Study

The DIM permeation/retention study was conducted in Franz diffusion cells (diffusion area of 3.14 cm^2^) using human skin as a barrier between donor and receptor compartments (CAAE: 27168719.4.0000.5346). The skin was positioned with the dermal layer facing the recipient medium, consisting of PBS pH 7.4 at 32 ± 0.5 °C (6 mL), and the stratum corneum facing the donor compartment (*n* = 6). Film fragments (1.0 cm^2^—corresponding to 106 µg of DIM, approximately) were placed in the donor compartment with the layer consisting of karaya gum facing the stratum corneum and hydrated with 200 µL of PBS pH 7.4 [54]. After 12 h of incubation, the film was removed, and the skin was submitted to tape stripping using 18 adhesive tapes (Scotch Brite^®^, 3M, São Paulo, Brazil). Afterward, the tapes were extracted in test tubes (3 tapes/tube) containing methanol (4 mL) using vortex (2 min) and ultrasound (15 min) for the quantification of the bioactive in the stratum corneum. Then, the epidermis and dermis were separated by a thermal bath (60 °C for 45 s). After this step, the epidermis was removed with the aid of a spatula, and the dermis was fragmented into small pieces, which were added to test tubes containing methanol (1 mL for epidermis and 2 mL for dermis) and subjected to vortex extraction (2 min) and ultrasound (15 min). All samples were filtered and analyzed by HPLC (Section 2.2) to determine the amount of DIM retained in each skin layer.

### 2.14. Antioxidant Activity

The antioxidant effect of polymeric films was evaluated through the scavenging capacity of the synthetic radical 2,2’-azinobis (3-ethylbenzothiazoline-6-sulfonic acid) (ABTS^+^), as described by Gehrcke et al. (2022) [37], with minor modifications. To initiate the process, an ABTS^+^ solution was generated by combining the ABTS^+^ stock solution (7 mM) with sodium persulfate (140 mM). This mixture was allowed to react for 12 h before the assay, and then it was diluted with phosphate buffer pH 7.0, resulting in a final ABTS^+^ concentration of 42.7 mM [55]. The film samples (0.5 cm^2^—corresponding to 53 µg of DIM) were placed in tubes containing 4 mL of ABTS^+^ solution, which were homogenized with a vortex (1 min) and incubated in the dark (30 min) (*n* = 3). An ABTS^+^ solution was maintained under the same reaction conditions and was used as a negative control. Blank samples containing film fragments and phosphate buffer pH 7.0 were also prepared in place of the radical solution. After incubation, the films were removed from contact with the solution, and its absorbance was measured at 734 nm (UV-1800 spectrophotometer, Shimadzu, Japan). The percentage of radical scavenging was calculated using Equation (4).

(4) $$SC\%=100-\frac{\left((AbsA-AbsB\right)\times 100)}{AbsC}$$

where SC% is the scavenging capacity in percentage, AbsA is the sample absorbance, AbsB is the blank absorbance, and AbsC is the negative control absorbance.

### 2.15. Biocompatibility Assays

The biocompatibility of films was evaluated by hemolysis assay and chorioallantoic membrane test (HET-CAM). These experiments were previously approved by the Research Ethics Committee of the Federal University of Santa Maria (CAAE: 27168719.4.0000.5346 and CEUA: 5428271020, respectively). The hemolysis assay was conducted in accordance with the Standard Practice for Assessment of Hemolytic Properties of Materials (ASTM, 2020) [56] (*n* = 3). Initially, 4.5 mL of blood was collected from a healthy volunteer, treated with 0.5 mL of citrate. Then, 2 mL of anticoagulated blood was centrifuged at 2000 rpm for 5 min, and the plasma was discarded. The resulting sediment was washed with saline 3 times to remove blood plasma completely. Then, the erythrocytes were resuspended in saline solution at a concentration of 10% (*v*/*v*). Film fragments (0.5 cm^2^) were inserted inside microtubes containing 700 µL of saline solution and equilibrated for 1 h. Afterward, 100 µL of resuspended erythrocytes was added to the tubes. The positive and negative controls were prepared using distilled water or saline solution, respectively. Also, blank samples were prepared, containing film fragments immersed only in saline solution instead of the erythrocyte suspension. Finally, the tubes were incubated (1 h at 37 °C), centrifuged (2000 rpm for 5 min), and the absorbance of the supernatant was measured in a spectrophotometer (540 nm). The percentage of hemolysis was calculated according to Equation (5).

The HET-CAM test was used to investigate the irritant potential of the films. Embryonated eggs were opened in the air chamber, and the chorioallantoic membrane (CAM) was carefully exposed. Film fragments (1.0 cm^2^) were positioned with the karaya gum layer facing the CAM, and phenomena such as vasoconstriction, hemorrhage, or coagulation were observed for a period of 300 s (*n* = 5) [57]. Negative and positive controls were 0.9% NaCl and 0.1 M NaOH, respectively. At the end of the exposure time, vascular events were photo-documented.

(5) $$Hemolysis=\frac{\left(AbA-AbB\right)}{AbC}\times 100$$

where AbA is the sample absorbance, AbB is the blank absorbance, and AbC is the positive control absorbance.

### 2.16. Statistical Analyses

The results are presented as the mean ± standard deviation (SD) or standard error of the mean (SEM). The normality of data distribution was assessed using the D’Agostino Pearson normality test. Subsequently, a *t*-test, or one or two-way analysis of variance (ANOVA), was conducted to evaluate the statistical significance, followed by the Newman−Keuls test based on the specific experimental design. Significance was defined as *p* < 0.05. GraphPad Prism^®^ version 6 statistical software was employed for all statistical analyses and figure generation.

## 3. Results and Discussion

The treatment of melanoma presents a challenge, as current therapies can cause adverse effects by attacking healthy cells in a non-specific manner [3]. In addition, regarding surgical removal, some regions affected by the lesions are difficult to remove with adequate safety margins, predisposing to tumor recurrence in the same location [58,59]. In this sense, some therapeutic alternatives for melanoma have been investigated, such as using less toxic natural compounds and nanostructured systems to reduce the systemic adverse effects that make the treatment unfeasible [4,21,60]. In addition, the development of formulations for application directly to the tumor site, such as gels, creams, and films, has been addressed to obtain a more significant amount of drug at the site of action, resulting in a more potent therapeutic effect and lower systemic adverse effects [18,20,31,32,61]. Our study aimed to create a bilayer film of pomegranate oil nanocapsules containing DIM, a bioactive that has been extensively researched for its potential to fight cancer.

### 3.1. Preparation and Characterization of Nanocapsule Suspensions

**Figure 1:**
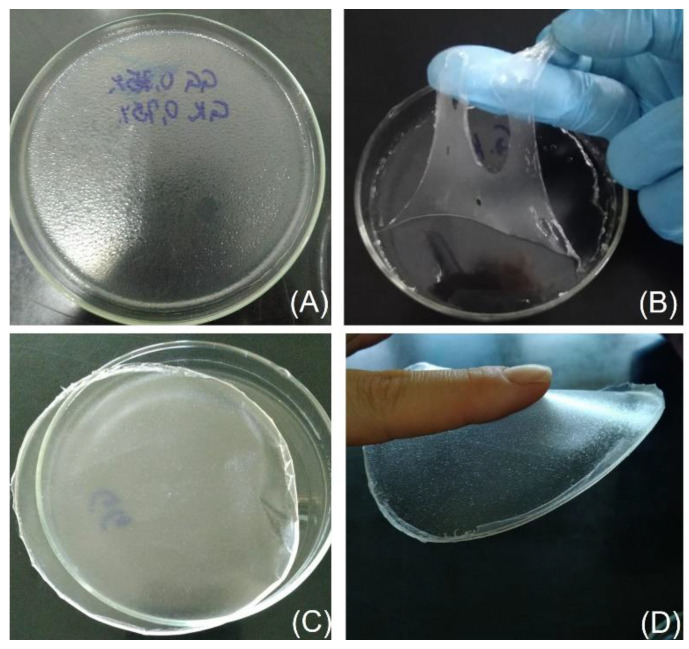
Images of films during pre-formulation tests. The first image shows the oily surface of films produced with nanocapsules stabilized with polysorbate 80 (**A**). The second image shows a fragile monolayer film made from karaya gum (**B**). The third image shows a bilayer film of gellan and karaya gums, stronger than the second film (**C**). The four images demonstrate the bioadhesive capacity of the film, present only in the qualitatively observed layer of karaya gum (**D**).

Films based on natural gums are characterized by hydrophilic matrices, which, by their nature, could hinder the incorporation of DIM due to its high lipophilicity [24,62]. Therefore, the adopted strategy involved the pre-encapsulation of the bioactive in polymeric nanocapsules, subsequently enabling an efficient DIM incorporation in the films. Initially, suspensions of polymeric nanocapsules were prepared using polysorbate 80, a surfactant with a highly hydrophilic−lipophilic balance. However, pre-formulation studies showed that these nanocapsules produced films with oily surfaces (Figure 1A). Based on this, polysorbate 80 was replaced by pullulan, a non-ionic polysaccharide already reported as a polymeric stabilizer for nanocapsule suspensions with high zeta potential in modulus and properties suitable for incorporation into films [25].

After preparation, the nanocapsule suspensions appeared opalescent and white, with no visible precipitates. Analysis showed parameters consistent with previous studies that used the same methodology for production [24,25,63]. The formulations had an acidic pH (around 4.8), nanoscale particle size (<160 nm), and a narrow size distribution (PDI < 0.07) (Table 1). Zeta potential evaluation confirmed the anionic nature of the nanocapsules with high modulus values (around −45 mV), consistent with previous studies that used this polysaccharide to stabilize nanosystems [25,27]. Bioactive concentration was close to theoretical values (0.96 mg/mL) for nanocapsules containing DIM, and encapsulation efficiency was high (close to 97%). Results suggest a uniform distribution of DIM in the lipophilic core of the nanocapsules, indicated by high values of EE% and similarity in size, PDI, and zeta potential parameters between NC-B and NC-DIM formulations (*p* > 0.05). This is particularly beneficial for effectively incorporating DIM, a highly lipophilic compound, into hydrophilic natural gum films [24].

### 3.2. In Vitro Antitumoral Activity

**Figure 2:**
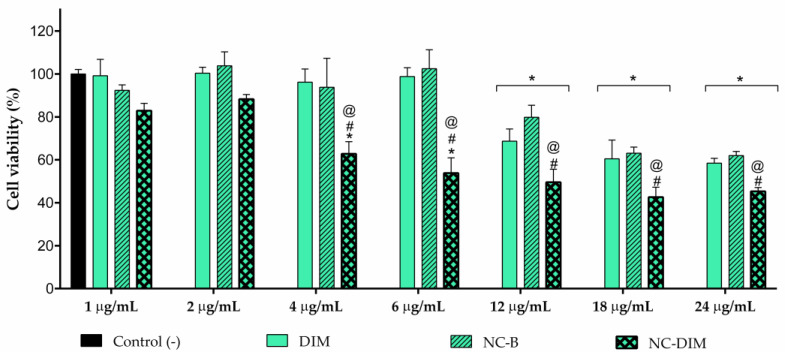
Evaluation of in vitro antitumoral activity against human melanoma cells (A-375). Mean ± standard deviation (*n* = 4). Statistical significance was assessed by one-way ANOVA followed by Newman−Keuls test. (*) *p* < 0.05: significant difference between negative control and the other groups. (#) *p* < 0.05: significant difference between NC-DIM and NC-B. (@) *p* < 0.05: significant difference between free DIM and NC-DIM.

Scientific studies have already shown that DIM has demonstrated evidence in cancer treatment, such as antioxidant action, invasion of neoplastic inhibition cells and metastatic angiogenesis, and induction of cell apoptosis in different types of neoplasia, including melanoma [6,7,9]. After evaluating the cytotoxicity of non-nanoencapsulated DIM and NC-B against A-375 cells, the results revealed that the bioactive compound required higher concentrations to reduce cell viability significantly (12, 18, and 24 µg/mL; Figure 2). In contrast, NC-DIM showed markedly increased cellular cytotoxicity compared to free DIM and NC-B, particularly at the lower concentrations (4 and 6 µg/mL; *p* < 0.05). These results were further emphasized by the IC_50_ values, which highlighted the substantial increase in the in vitro antitumor efficacy of DIM through its nanoencapsulation (>24.00 µg/mL of free DIM vs. 2.89 µg/mL of nanocapsules; Table 1).

In this context, the present study underscores the potential of DIM nanoencapsulation to significantly enhance its antitumor efficacy against melanoma cells, thereby offering a promising avenue for neoplasm therapy. This finding corresponds with similar research where polymeric nanocapsules enhanced the effectiveness of pharmaceutical agents against melanoma [64,65]. Notably, previous investigations have also highlighted the advantageous role of vegetable oils, such as pomegranate oil, within the oily core, in amplifying the antitumor potential of compounds [66,67]. Consequently, the nanocapsules generated here exhibit promise as an intermediate product within the spectrum of film production, with the potential to be harnessed for anti-melanoma therapy.

### 3.3. Pre-Formulation Studies

The successful development of films is based on the proper selection of film-forming agents and plasticizer concentrations when necessary [33]. Initially, optimizations were sought in monolayer films composed only of suspensions of nanocapsules and karaya gum based on previous studies of films with this gum [44,45,68]. Gum concentrations above 0.75% (*w*/*v*) resulted in very viscous polymeric dispersions, making transferring difficult for a drying surface. Furthermore, the films obtained were brittle, rigid, and adherent to the Petri dish. In this sense, different concentrations of glycerol (1 and 2% *w*/*v*) as a plasticizer were evaluated, but despite increasing the elasticity and facilitating the detachment of the films, they remained fragile (Figure 1B). Based on this, other possible plasticizers (PEG 400 and sorbitol) were also tested, which did not change the fragility of the films already identified with glycerol. In our research group, films of gellan gum containing silibinin-loaded polymeric nanocapsules had recently been developed. These films were resistant and had suitable properties for cutaneous application [24]. Given this, the hypothesis arose to create a bilayer film with a combination of karaya gum and gellan gum, to obtain a film with adequate mechanical resistance.

This approach allowed us to obtain films resistant enough to be removed from the Petri dish and applied to the skin (Figure 1C). Bilayer films maintain the intrinsic properties of the polymeric materials in each layer, and the first layer, composed of gellan gum, provides the necessary mechanical strength [34,36,37]. Additionally, qualitative analysis revealed a high bioadhesive potential on the karaya gum face of the bilayer film (Figure 1D). In contrast, the gellan gum face remained non-adherent, indicating that the properties of the isolated polymeric materials were maintained in their respective polymeric layers [37].

**Figure 3:**
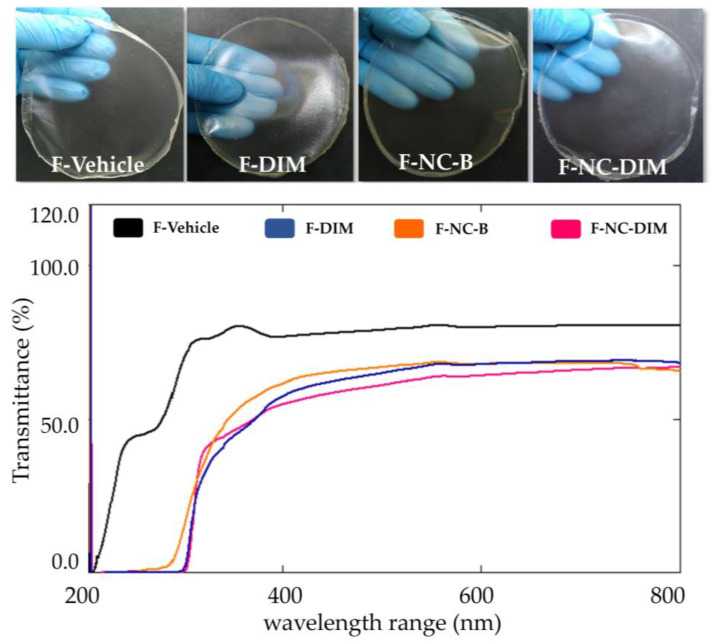
Macroscopic appearance and UV–Vis spectra of developed bilayer films.

After drying, the films showed remarkable flexibility and strength, making them easily handled and adaptable to the application site. Furthermore, regardless of the presence of nanocapsules, the films maintained a homogeneous appearance and were transparent (Figure 3). The high transmittance, recorded above 65% in the UV–Vis scan, proved the transparency of the films [24] (Figure 3). This characteristic is particularly relevant for cutaneous use, especially considering its possible application in post-surgical lesions for melanoma removal, allowing the visualization of the injured tissue through the film, and avoiding the need to remove it. When comparing the different films in the transmittance spectrum, a reduction was noted in the vehicle film after the incorporation of free DIM or nanocapsules. Similar results were obtained by other studies that developed films containing nanosystems, and this effect was attributed to the ability of colloidal systems to scatter light [24,46,69]. Regarding the film containing the free bioactive, the reduction in transmittance may be suggestive of crystals of the bioactive, considering its lipophilicity and insertion in a hydrophilic matrix [10]. This finding is reinforced by the SEM images where the presence of structures characteristic of crystals was observed on the face of the gellan gum. On the other hand, this limitation can be circumvented by previously encapsulating DIM in nanocapsules since the bioactive can be deposited in the oil core and then homogeneously incorporated into the films [24].

### 3.4. Film Characterization

The values for thickness, particle size, DIM content homogeneity, and weight homogeneity are shown in Table 2. The thickness of films is directly related to comfortable administration, as they tend to be relatively thinner than conventional patches for cutaneous use [70]. The thickness values found in our study were between 178 and 480 μm, indicating that the films developed are considered thin and are similar to other polymeric films produced for cutaneous use [25,33,70]. Notably, F-DIM showed greater thickness (*p* < 0.05), which may be linked to the excessive use of PEG 400 in this preparation to allow DIM to be served in the films. It is known that PEG 400 can act as a plasticizer in films, interposing itself between the polymeric chains, and reducing the intermolecular forces between the chains, resulting in greater thickness [33,71]. In contrast, thinner films were obtained by placing DIM in polymeric nanocapsules, which may represent greater comfort in the cutaneous administration of these formulations [25,33].

Films have advantages over semi-solid dosage forms for cutaneous use, including dose accuracy. This accuracy is directly related to the film’s weight and active substance content, which must be homogeneous [33,37]. The produced films showed reduced weight variation (<2 mg). Furthermore, considering the DIM content, both films (F-DIM and F-NC-DIM) showed values close to 106 µg/cm^2^, with reduced variation (SD < 2 µg) (Table 2). In this sense, the developed formulations had satisfactory content and weight characteristics that could result in dose homogeneity when administered in the cutaneous tissue, as evidenced by the low standard deviation value.

When investigating the granulometric analysis of nanostructured systems, it is common to observe whether the nanometric scale is maintained after it is incorporated into final dosage forms [25,32]. The mean particle diameter increased after incorporation into films (around 326 nm for F-NC-B and F-NC-DIM) (*p* < 0.05) (Table 2). This aligns with other studies that found increased size and PDI when nanosystems were carried in hydrogels or films of natural or synthetic polymers [32,72]. The deposition of karaya gum chains on the surface of polymeric nanocapsules may occur during the drying process of films. However, studies indicate that this size increase does not usually affect the pharmacological performance of nanotechnology-based films when applied to the skin [25].

### 3.5. Scanning Electron Microscopy

**Figure 4:**
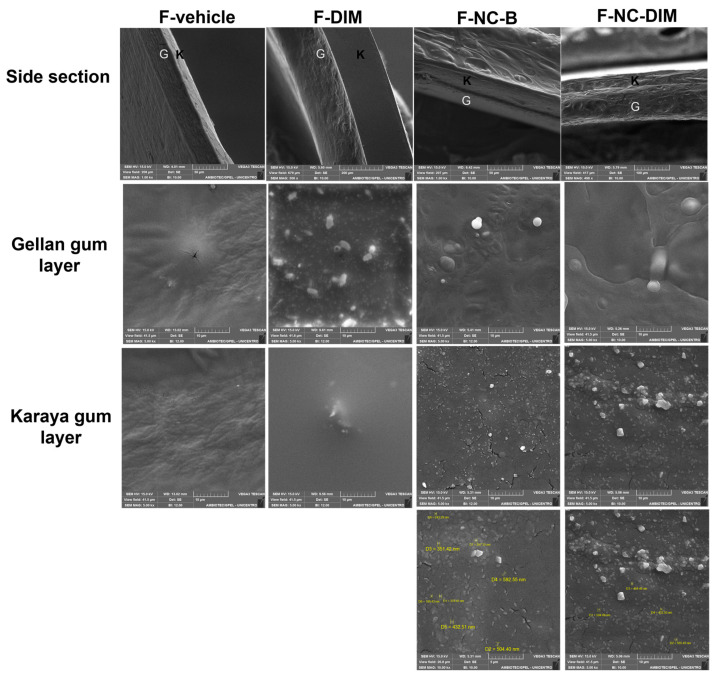
Scanning electron microscopy images were obtained for the different films in their different layers (karaya or gellan gum), and side sections. The K indicates the karaya gum layer, and G indicates the gellan gum layer in the side section of polymeric films.

During morphological evaluation, two polymeric layers were detected in the lateral sections of the films (Figure 4), confirming layer maintenance after drying. Structurally, this organization is relevant, as it allows the gums to remain isolated in their respective layers, maintaining their intrinsic properties, such as the adhesion of the karaya gum and the low adherence and resistance of the gellan gum [44,45]. Additionally, during the evaluation of the surface of the films, the presence of structures suggestive of bioactive crystals in the layer of gellan gum in F-DIM was evidenced. Considering that DIM is highly lipophilic, this may have occurred due to incompatibility with the hydrophilic matrix of natural gums [63]. Furthermore, the presence of crystals may be related to the decrease in transmittance levels observed for F-DIM during UV–Vis scanning. In this study, the data emphasize the necessity of producing polymeric nanocapsules that contain DIM. These nanocapsules containing DIM with high encapsulation efficiency can then be placed in polymeric films. This, in turn, prevents the formation of precipitates by keeping the DIM molecularly dispersed inside the oily core [24].

Regarding the surface of karaya gum in nanocapsule films (F-NC-B and F-NC-DIM), it presented spherical structures varying between 300 and 500 nm in size. Such images may indicate the presence of nanocapsules on the surface, which may be covered by the a film-forming agent, thus justifying the increase in size and corroborating the data from photon correlation spectroscopy [32,72].

### 3.6. Mechanical Properties

When creating films for cutaneous application, it is essential to investigate their mechanical properties. This helps to determine their ability to resist rupture and adapt during application. While films must be flexible enough to adapt, they must maintain their structure for adequate dose homogeneity [25,73]. The films showed deformation values ranging between 3.3% and 6.0%, with no significant changes regardless of their composition (*p* > 0.05). However, when it comes to tensile strength, it was found that F-DIM had lower values compared to the other films (*p* < 0.05). This outcome is consistent with the data obtained for Young’s modulus, as F-DIM also had lower values (*p* < 0.05) (Table 2). In contrast, the films constituted by nanocapsules (F-NC and F-NC-DIM) showed similar tensile strength and Young’s modulus to the F-vehicle (*p* > 0.05).

Tensile strength and Young’s modulus are indicators of a film’s resistance to deformation and the force required to break it, respectively [74]. Excessive plasticizers can make films more fragile by reducing intermolecular interaction between polymer chains [33]. This explains the fragility of F-DIM, which has an excess plasticizer for bioactive solubilization. However, F-NC-DIM, which has nanoencapsulated DIM, did not affect its strength or Young’s modulus. This suggests that inserting DIM into nanostructured systems can result in stronger films. The tensile strength values of the obtained film were similar to those of other gellan gum films, indicating that the strength of films can come from this gum layer [24,37].

### 3.7. Water Contact Angle

**Figure 5:**
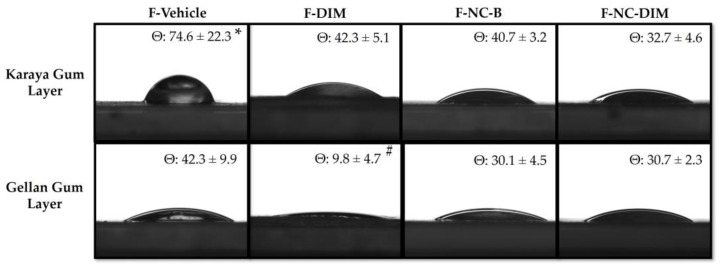
Representative image of the contact angle evaluations of the different films in both layers (gellan gum or karaya gum). The results are expressed as mean ± standard deviation (*n* = 3). Statistical significance was assessed by one-way ANOVA followed by Newman−Keuls test. (*) *p* < 0.05: significant difference between F-vehicle and the other films. (#) *p* < 0.05: significant difference between NC-DIM and the other films.

The contact angle is a commonly used measure to determine the affinity between water-based fluids and the surface of films. An angle of less than 90° indicates greater wettability, whereas higher angles suggest greater hydrophobicity [24]. Based on the obtained contact angle values, it can be inferred that the films possessed hydrophilic surface characteristics since all values were less than 90°, indicating they are suitable for absorbing fluids from injured tissue (Table 2; Figure 5).

Regarding the face of gellan gum, lower contact angle values were observed for F-DIM compared to the other films (*p* < 0.05), which may again be related to the excess PEG 400 used in preparing this formulation. Cervi et al. (2021) showed that when developing polymeric films of pullulan using PEG 400, this plasticizer leaked onto the underside of the films. This finding was related to the contact angle data observed for the underside of the films, which showed reduced angles.

Regarding the contact angle on the face of karaya gum, the surface of the vehicle film was less hydrophilic when compared to the other films (*p* < 0.05). This fact was due to the acetyl groups present in the structure of the karaya gum that confer hydrophobicity to this polymer [43]. In contrast, the presence of nanocapsules on the karaya gum face of the films (F-NC-B and F-NC-DIM) resulted in a more hydrophilic surface, which may have been related to the use of pullulan as a surface-active agent in stabilizing the nanocapsules, since this is a polysaccharide with a hydrophilic character [75]. With respect to the intended application, the hydrophilic surface of the films obtained can be attractive, as it favors the absorption of possible fluids from injured tissue, as is the case of ulcerative melanoma lesions or in the healing stages after tumor surgical removal [76].

### 3.8. Swelling Index

Previous research has shown that natural gum films have a high capacity for absorbing aqueous fluids [44,45]. The ability to absorb fluids is usually linked to the number of groups in the polymer chain that can form hydrogen bonds. Furthermore, plasticizers added to films can modify them through intermolecular interactions with polymeric chains [33]. Table 2 shows the swelling results of the tested films, which were 809 ± 66%, 284 ± 25%, 776 ± 21%, and 768 ± 49% for F-vehicle, F-DIM, F-NC-B, and F-NC-DIM, respectively. It was observed that the F-DIM presented significantly lower swelling values (*p* < 0.05) when compared to the other films. This indicates that although F-DIM has a hydrophilic surface, it has limited ability to absorb aqueous fluids, possibly due to excess interactions between PEG 400 and hydroxyl groups, resulting in fewer groups available to interact with the water molecules, leading to less hydration [33]. On the other hand, films made from nanoencapsulated DIM showed high swelling and were more compatible with fluid absorption from skin lesions [37].

### 3.9. Bioadhesive Strength

**Figure 6:**
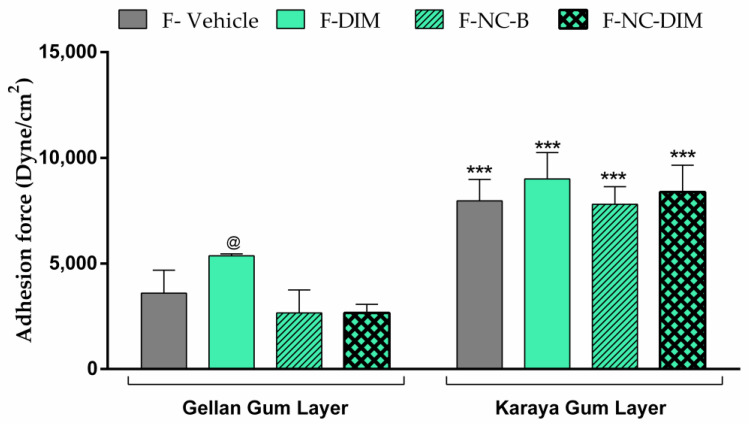
Evaluation of the bioadhesive strength of films on the skin. The results are expressed as mean ± standard deviation (*n* = 3). Statistical significance was assessed by one-way ANOVA followed by Newman−Keuls test. (***) *p* < 0.001: significant difference between gellan gum layer and karaya gum layer for the same film. (@) *p* < 0.05: significant difference between F-DIM and the other films in the gellan gum layer.

The bioadhesive potential of the formulations is a crucial factor for the therapeutic effect, ensuring permanence in the application site for the necessary time [37]. Films must have adequate adhesion to the skin without adhesive tapes that cause skin irritation [70]. Based on the bioadhesion evaluation of films, it was discovered that the formulations had a higher bioadhesive strength in the karaya gum layer, and these were similar to each other (*p* > 0.05; Figure 6). This result is essential since this layer would be in contact with the skin and should have a high bioadhesive ability.

On the other hand, the layer of the gellan gum, which would face the external environment, must not be bioadhesive. Films containing polymeric nanocapsules had reduced adhesion in this layer, unlike what happened with F-DIM (*p* < 0.05). This fact may be associated with the excess PEG 400 in F-DIM, which was used to solubilize the bioactive. It is known that PEG 400 can act as a plasticizer in films and that excess PEG 400 in these films can lead to a significant relaxation of the polymeric chains, making the films thicker and more fragile [33]. Thus, it can be suggested that the excess of PEG resulted in a greater relaxation of the gellan gum chains in the F-DIM, which may have exposed hydroxyl groups of this polymer, allowing more significant interaction with the cutaneous tissue [33,77]. Thus, films made of nanoencapsulated DIM (F-NC-DIM) are more promising than those made of the free bioactive (F-DIM), maintaining high adhesion on the face of karaya gum and low adhesion on the layer of gellan gum, preventing adherence to clothes, and external dirt.

### 3.10. In Vitro DIM Release

**Figure 7:**
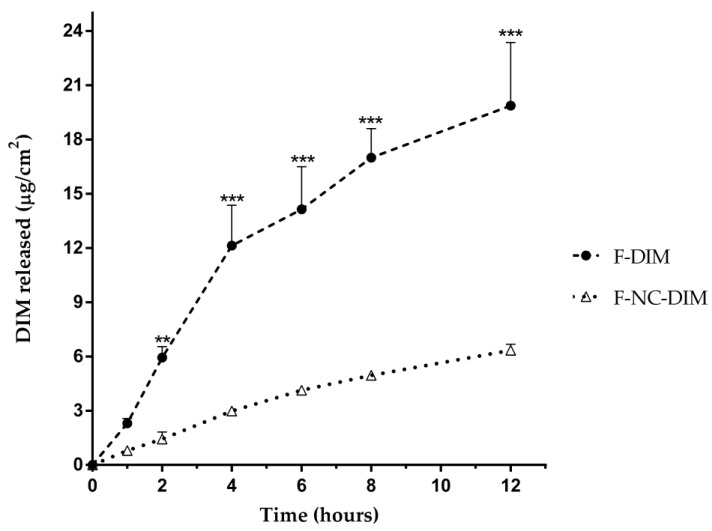
In vitro release profile of DIM from film containing non-nanoencapsulated DIM (F-DIM) or nanoencapsulated DIM film (F-NC-DIM). Statistical significance was assessed by two-way ANOVA followed by Newman−Keuls test. (**) *p* < 0.01; (***) *p* < 0.001: significant difference between F-DIM and F-NC-DIM.

According to various studies, it has been observed that the delivery of nanosystem active substances in films leads to a controlled drug release [24,78,79]. Our study also produced similar results, highlighting the controlled release of DIM when it was nanoencapsulated. After 12 h, F-NC-DIM released 6.3 ± 0.3 µg/cm^2^ of DIM to the receptor medium, while F-DIM released 19.9 ± 3.5 µg/cm^2^ (Figure 7). The total amount released was three times lower for F-NC-DIM, and composed of nanocapsules (*p* < 0.05). In terms of percentage, the amount of DIM transferred to the medium was 18.8 ± 1.0% for F-NC-DIM and 58.8 ± 10.4% for F-DIM. These data corroborate other authors who highlight the release-control role played by karaya gum. Thus, this polysaccharide may have resulted in a controlled release even in films where the bioactive was incorporated in its free form [80,81]. In the case of nanotechnology-based films, the more expressive release control may be due to the effects known for karaya gum, in addition to the presence of ethylcellulose present in the nanocapsules since this polymer is also known to result in controlled release [15,82].

The release kinetics analyses demonstrated that both films showed zero-order release kinetics (regression coefficients of 0.9020 and 0.9694 for F-DIM and F-NC-DIM, respectively) (Table 3). These data indicate that DIM is transferred in constant form to the medium over time, regardless of the amount of bioactive present in the matrix [83,84]. Additionally, the polymeric films proved adequate to the Higuchi model (regression coefficient of 0.9748 and 0.9969 for F-DIM and F-NC-DIM, respectively), confirming that the release process occurred predominantly by controlled diffusion [85]. These results are significant, as the gradual release of the bioactive from the film can have a prolonged biological effect on the skin tissue. Additionally, the controlled release of DIM, combined with the bioadhesive potential of the films, can reduce the need to change the film applied to the skin frequently. This can result in greater comfort for patients suffering from ulcerative lesions or those who have surgically removed tumor tissue [24,76,79].

### 3.11. DIM Skin Permeation/Penetration Study

**Figure 8:**
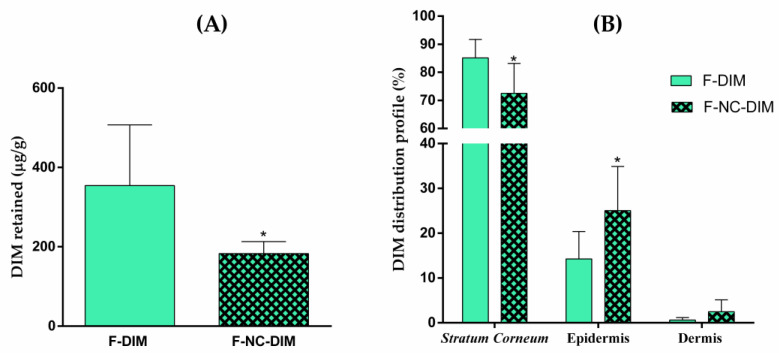
(**A**) represents the cumulative amount of DIM in the skin after 12 h of incubation with bilayer films containing non-nanoencapsulated DIM (F-DIM) or nanoencapsulated DIM (F-NC-DIM). (**B**) represents the skin permeation profile of film containing non-encapsulated DIM (F-DIM) or nanoencapsulated DIM (F-NC-DIM). The results are expressed as mean ± standard deviation (*n* = 6) for the unpaired *t*-test at each skin layer or retained total. (*) *p* < 0.05: significant difference between F-DIM and F-NC-DIM.

The results of the permeation experiment demonstrated that the amount of DIM retained in the skin was higher for F-DIM (354.43 ± 152.68 µg/mL) than for F-NC-DIM (182.89 ± 30.07 µg/mL) (*p* < 0.05) (Figure 8A). This result was attributed to the controlled release profile of DIM from the nanotechnology-based film, as observed in the in vitro release experiment. Studies have shown that using nanocapsules as a controlled release system for active substances can reduce skin permeation [24,86].

Subsequently, a relative distribution profile of DIM in the different layers of the skin was obtained, taking the total permeation as 100% (Figure 8B). The percentage distribution values for the F-DIM were 85.13 ± 6.58%, 14.25 ± 6.12%, and 0.62 ± 0.57% for the stratum corneum, epidermis, and dermis, respectively. Regarding the F-NC-DIM, the percentages were 72.5 ± 10.64%, 25.04 ± 9.84%, and 2.47 ± 2.68% for the stratum corneum, epidermis, and dermis, respectively. In a comparative form, F-NC-DIM resulted in a much better distribution of DIM in the viable epidermis (*p* < 0.05). This change in the distribution of the active substance was expected since polymeric nanocapsules may increase the possibility of the drug reaching viable layers of the skin [18,32,87]. Moreover, this finding is relevant because it indicates that the nanoencapsulation of DIM resulted in an increased direction of the bioactive in the layer where the neoplastic process originates since the melanocytes are found in the basal layer of the epidermis [3]. Therefore, nanoencapsulation of the bioactive can aid in delivering it to the therapeutic layer.

### 3.12. Antioxidant Activity

**Figure 9:**
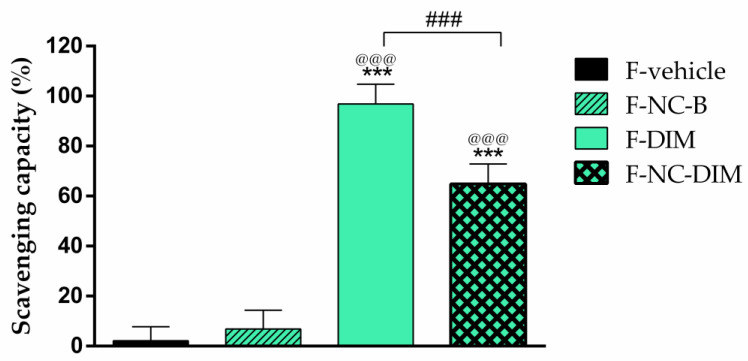
Scavenging activity of films against the ABTS^+^ radical. The results are expressed as mean ± standard deviation (*n* = 3). Statistical significance was assessed by one-way ANOVA followed by Newman−Keuls test. (***) *p* < 0.001: significant difference between F-vehicle and the other films. (@@@) *p* < 0.001: significant difference between F-NC-B and the other films. (###) *p* < 0.001: significant difference between F-DIM and F-NC-DIM.

Oxidative stress emerges as a factor closely related to DNA damage, pro-inflammatory processes, and carcinogenesis [7,88]. Given the already recognized antioxidant and antitumor ability of DIM and the interest in applying the films developed in melanoma therapy, the antioxidant activity of polymeric films was determined against the synthetic radical ABTS^+^. The percentages of ABTS^+^ radical neutralization were 5.7 ± 5.6%, 6.8 ± 7.5%, 99.8 ± 7.9%, and 64.8 ± 8.0% for F-vehicle, F-NC-B, F-DIM, and F-NC-DIM, respectively (Figure 9).

Notably, F-DIM and F-NC-DIM differed (*p* < 0.05), showing greater radical scavenging ability than F-vehicle and F-NC-B. The results highlighted that the association of DIM, either in free or nanoencapsulated form, in polymeric films exhibits a remarkable neutralizing power about the ABTS^+^ radical, evidencing the preservation of the antioxidant potential already reported for the bioactive after its incorporation in the films [15]. Consistent results were obtained in studies involving other polymeric films loaded with antioxidant substances [62,78]. When comparing the F-DIM and F-NC-DIM films, a greater neutralization capacity was observed in the film containing DIM in its free form (*p* < 0.05). This finding is possibly linked to the controlled release previously observed in the in vitro release assay of F-NC-DIM.

### 3.13. Biocompatibility Tests

**Figure 10:**
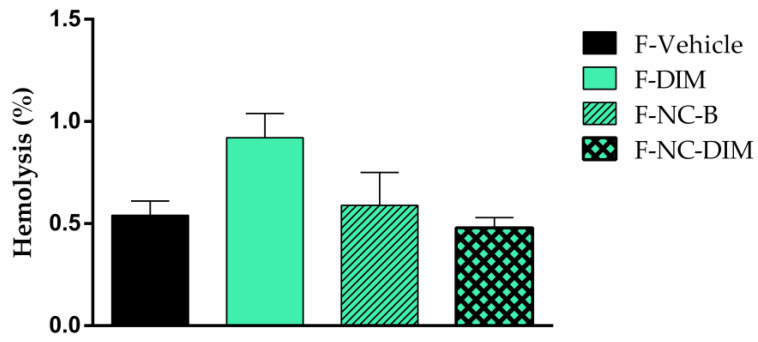
Evaluation of the hemolytic potential of films. The results were expressed as mean ± standard deviation (*n* = 3).

When treating melanoma, common therapies often cause skin irritation, photosensitivity, and redness [89,90]. To reduce these side effects, researchers are exploring new therapeutic approaches. In this sense, the films were submitted to hemolysis and HET-CAM tests to investigate their biocompatibility. The percentage of hemolysis for F-vehicle, F-DIM, F-NC-B, and F-NC-DIM were 0.54 ± 0.07%, 0.92 ± 0.12%, 0.59 ± 0.16%, and 0.48 ± 0.05%, respectively (Figure 10). These rates were similar to the negative control (0.85 ± 0.27%) (*p* > 0.05). According to the evaluation of the hemolytic properties of materials [56], materials with hemolysis lower by up 2% can be considered non-hemolytic. This result was in line with previous predictions, given that previous research that addressed the creation of films based on karaya gum or gellan had already documented the absence of any hemolytic potential [37,91].

**Figure 11:**
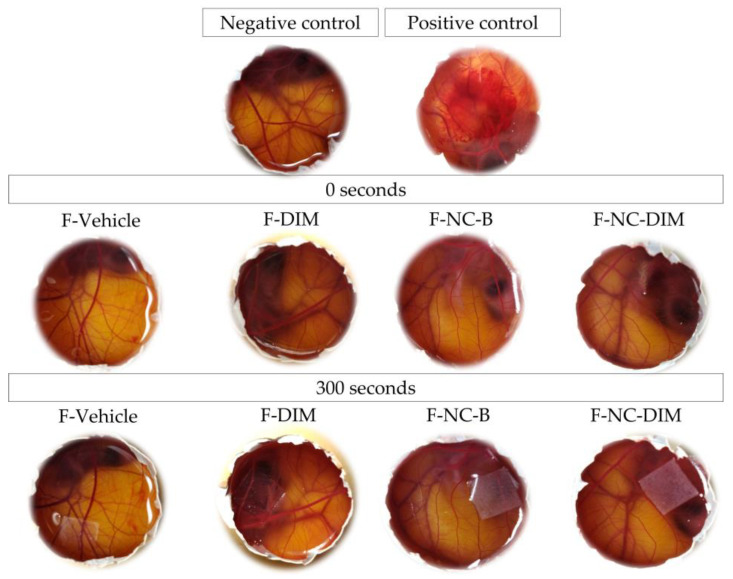
Representative images of the chorioallantoic membrane (CAM) test after applying different films, negative (0.9% NaCl) and positive (0.1 M NaOH) control.

In the HET-CAM assay, it was found that only the group treated with the positive control (0.1 M NaOH) showed irritation events (hemorrhage and coagulation) after 300 s of exposure. However, none of the tested films caused vascular events such as vasoconstriction, hemorrhage, or coagulation, similar to the negative control (NaCl 0.9%), classifying the films as non-irritating (Figure 11) [57]. The results corroborate the research conducted by Gehrcke et al. (2021), who also evaluated polymeric films containing ethylcellulose nanocapsules using the same methodological approach. Furthermore, the biocompatible safety and absence of toxicity of karaya gum have been reiterated in previous studies [92,93]. Thus, polymeric films were found to be non-irritating and non-hemolytic [56,57]. This suggests they could provide a safer alternative for cutaneous melanoma therapy with fewer unwanted effects.

## 4. Conclusions

In summary, the results achieved in this study provide an encouraging perspective for the cutaneous application of polymeric films containing DIM as an innovative strategy in the treatment of melanoma. Nanoencapsulation of the bioactive has holds promise for the potentiation of the antitumor effect in vitro. The films’ remarkable transparency, homogeneity of weight, consistent DIM content, and optimal mechanical strength support their suitability as highly promising formulations for cutaneous administration. The more significant bioadhesive potential of the karaya gum layer and the lower adherence of the gellan gum layer were proven to be an intelligent approach to ensuring prolonged adhesion in affected areas while minimizing discomfort associated with unwanted attachment to the external environment. The controlled release profile of DIM from the nanocapsule films represents a remarkable achievement, suggesting that regular cutaneous administration can maintain consistent levels of the active substance for longer. In addition, much better distribution of the bioactive in the viable epidermis was observed in the skin permeation, which is interesting for melanoma therapy since this is usually the initial development layer of tumor lesions.

Furthermore, the maintenance of the antioxidant potential of the bioactive and the absence of hemolytic and irritating effects consolidate the safety and tolerability of the films as a viable option for treating melanoma by the cutaneous route. This represents remarkable progress compared to conventional treatments that often have debilitating side effects. In summary, the polymeric films developed in this study offer a promising horizon for the cutaneous therapy of melanoma. As we proceed to the following stages, conducting preclinical studies may provide a complete understanding of the efficacy and safety of these innovative films, which could open the door to potential clinical applications.

## Figures and Tables

**Table 1:** Characterization of NC-DIM and NC-B formulations and IC_50_ values for free DIM, NC-B, and NC-DIM.

	Free DIM	NC-B	NC-DIM
pH	-	4.8 ± 0.1	4.8 ± 0.1
Particle size (nm)	-	157 ± 2	154 ± 1
Polydispersity index	-	0.07 ± 0.01	0.07 ± 0.00
Zeta potential (mV)	-	−45.0 ± 2.0	−54.0 ± 6.0
DIM content (mg/mL)	-	-	0.96 ± 0.03
Encapsulation efficiency (%)	-	-	97.0 ± 1.0
A-375 cells IC_50_ (µg/mL)	>24	>24	2.89

The results are expressed as mean ± standard deviation (*n* = 3)—unpaired Student’s *t*-test. There was no significant difference between NC-B and NC-DIM (*p* > 0.05).

**Table 2:** Results of bilayer films’ characterization.

	F-Vehicle	F-DIM	F-NC-B	F-NC-DIM
Weight homogeneity (mg/cm^2^)	18.3 ± 0.4	48.8 ± 0.9	21.6 ± 1.2	21.2 ± 0.7
Drug content homogeneity (µg/cm^2^)	-	101.5 ± 1.8	-	105.6 ± 1.6
Thickness (µm)	208 ± 29	480 ± 12 ^+^	210 ± 12	178 ± 22
Particle size (nm)	504 ± 142	-	326 ± 51 *	326 ± 43 *
Polydispersity index	0.6 ± 0.2	-	0.5 ± 0.1	0.5 ± 0.1
Swelling index (%)	809 ± 66	284 ± 25 ^+^	776 ± 21	768 ± 49
Water contact angle (karaya gum layer)	74.6 ± 22.3	42.3 ± 5.1 *	40.7 ± 3.2 *	32.7 ± 4.6 *
Water contact angle (gellan gum layer)	42.3 ± 9.9	9.8 ± 4.7 ^+^	30.1 ± 4.5	30.7 ± 2.3
Tensile strength (MPa)	2.9 ± 0.80	0.9 ± 0.25 ^+^	2.6 ± 0.62	3.5 ± 0.47
Elongation (%)	5.3 ± 1.9	5.6 ± 1.4	3.3 ± 0.4	6.0 ± 1.1
Young’s modulus (MPa)	59.3 ± 22.3	16.5 ± 6.01 ^+^	78.5 ± 18.9	58.9 ± 2.66

The results are expressed as mean ± standard deviation (*n* = 3). Statistical significance was assessed by one-way ANOVA followed by Newman−Keuls test. (*) *p* < 0.05: significant difference between F-vehicle and the other films. (^+^) *p* < 0.05: significant difference between NC-DIM and the other films.

**Table 3:** Regression coefficients obtained for the different mathematical models applied to the in vitro release profile of polymeric films.

Mathematical Models	F-DIM	F-NC-DIM
Zero order	0.9020	0.9694
First order	0.7157	0.8299
Second order	0.5068	0.6355
Higuchi model	0.9748	0.9969

## Data Availability

Not applicable.
